# Supplementary material for: Potential Role of Hydroxyapatite Nanocrystalline for Early Diagnostics of Ovarian Cancer
Source: Diagnostics (Basel). 2021 Sep 22;11(10):1741. doi: 10.3390/diagnostics11101741 (PMC8534774; doi:10.3390/diagnostics11101741)
Supplement: Supplementary file 1 [file diagnostics-11-01741-s001.zip › diagnostics-1359281-supplementary.pdf]

Table S1. Control group. Ovarian tumors without calcifications

Case	Age of women	TNM			Tumor diameter (larger size), mm
		T	N	M	
**1**	60	T3	N1	M0	21
**2**	50	T4	N1	M1	80
**3**	33	T1	N1	M0	20
**4**	48	T3	N1	M0	90
**5**	39	T3	N1	M0	70
**6**	64	T2	N1	M0	60
**7**	77	T2	N1	M0	60
**8**	68	T2	N1	M1	15
**9**	48	T2	N0	M0	20
**10**	64	T1	N0	M0	50
**11**	33	T1	N0	M0	20
**12**	56	T3	N1	M0	30
**13**	55	T2	N0	M0	25
**14**	66	T1	Nx	M0	100
**15**	49	T1	Nx	M0	20
**16**	44	T2	N1	M0	45
**17**	51	T3	N1	M0	5
**18**	39	T3	N1	M0	10
**19**	57	T3	N1	M0	35
**20**	36	T2	N0	M0	10
**21**	46	T3	N1	M0	20
**22**	49	T3	N1	M0	6
**23**	52	T1	Nx	M0	60
**24**	45	T1	N0	M0	10
**25**	59	T3	N1	M1	10
**26**	73	T1	N0	M0	40
**27**	54	T3	N1	M0	15
**28**	62	T1	Nx	M0	80
**29**	45	T3	N1	M0	120
**30**	63	T2	N0	M0	45

Notes. T – tumor size, N – metastases in lymphatic nodules, M – distant metastases
